# Hot Cracking Behaviors of Mg-Zn-Er Alloys with Different Er Contents

**DOI:** 10.3390/ma16093546

**Published:** 2023-05-05

**Authors:** Yaohong Liu, Zhaohui Wang, Shubo Li, Ning Ding, Ke Liu, Wenbo Du

**Affiliations:** Faculty of Materials and Manufacturing, Beijing University of Technology, Beijing 100124, China

**Keywords:** Mg-Zn-Er alloy, solidification process, hot cracking behaviors, microstructure, permeability characteristics

## Abstract

The hot cracking behaviors of Mg-5Zn-*x*Er (*x =* 0.83, 1.25, 2.5, 5 wt.%) alloys are investigated by optimized hot cracking experimental apparatus, optical microscope, and scanning electron microscope, such as contraction behaviors, feeding behaviors, and permeability characteristics. It is found that the solid phase fraction at hot crack initiation and within the freezing range both increased with increasing Er contents up to 2.5 wt.% and then decreased at 5 wt.% Er content. The Mg-5Zn-5Er alloy exhibits the lowest solid phase fraction (87.4%) and a reduced freezing range (74.2 °C), which leads to more effective liquid feeding in the latter stages of solidification. Combined with the grain size, the permeability of the mushy zone, and fracture morphology, the overall permeability is optimal in the Mg-5Zn-5Er alloy, which is beneficial for feeding the cavities and micro-pores. Meanwhile, a large amount of W phase precipitated by the eutectic reaction (L→α-Mg + W phase), which facilitates healing of the incurred cracking. Conversely, the Mg-5Zn-2.5Er alloy shows inferior feeding ability due to the lowest solid phase fraction (98.3%), wide freezing range (199.5 °C), and lowest permeability. Therefore, the Mg-5Zn-2.5Er alloy exhibits maximal hot cracking susceptibility, and the Mg-5Zn-5Er alloy exhibits minimal hot cracking susceptibility. This work provides guidance for improving the hot cracking resistance of cast Mg-Zn-Er alloy and enables an understanding of the hot cracking behaviors of Mg-Zn-RE alloys.

## 1. Introduction

Magnesium alloys have attracted considerable attention in the electronic, aeronautical, and aerospace industries due to their low density, high specific strength, high specific stiffness, excellent damping, and recyclability [1,2]. The casting process has outstanding dimensional accuracy, a short processing cycle, and high production efficiency and has become an important processing method for magnesium alloy parts (approximately 90%) [3,4]. Casting properties are vital in casting production, which directly affects the qualities of the products, such as hot cracking susceptibility [5].

The hot cracking behaviors of magnesium alloys during solidification are complex and influenced by many factors, including alloy constitution, phase composition, grain size, solidification path, etc. [6,7,8,9,10,11]. Until now, scholars have carried out a series of investigations on the hot cracking behaviors of cast magnesium alloys. Du et al. [12] studied the effects of Sb, Gd, and the combined addition of Sb and Gd on the hot-cracking behaviors of Mg-Al-Ca alloy and found that the hot cracking susceptibility of the Mg-5Al-3Ca alloy was minimal due to the vulnerable temperature range and increased coherency solid fraction via the addition of 0.5 wt.% Sb and 0.5 wt.% Gd. The hot cracking susceptibility of the Mg-10Zn alloy with the addition of different Al elements (0, 2, 5, 7 wt.%) was investigated using the experimental setup of constrained rod casting by Vinodh et al. [13]. The results suggested that the hot cracking susceptibility of the Mg-10Zn-*x*Al alloy decreased as the Al content increased, which was ascribed to the following reasons: (i) reduced freezing range; (ii) higher liquid fraction at the late solidification; (iii) the effect of grain refinement; and (iv) the formed cavities during solidification could be healed by the addition of eutectic liquids. Zhou et al. [14] demonstrated that the hot cracking susceptibility of the Mg-7Zn-*x*Cu (*x* = 0, 1, 2, 3 wt.%) alloy decreased with increasing Cu content due to the decreased effective stress, thick liquid film covering the dendrites and reduced freezing range. Wei et al. [15] reported that the hot tearing susceptibility of Mg-7Gd-5Y-0.5Zr (wt.%) linearly decreased by adding Zn from 3 to 5 to 7 wt.%. This was mainly attributed to the increasing dendrite coherency temperature of the α-Mg (*T*_coh_), the reduction of the intergranular feeding liquid channel, and the decreased solidification shrinkage force. However, Song et al. [16] showed that the Mg-0.5Ca (wt.%) alloy exhibited higher hot cracking susceptibility with the addition of Zn (0.5, 1.5, 4.0 wt.%). Summarizing the current progress, it can be concluded that the alloying elements had a critical influence on the hot cracking behaviors of the casting Mg alloy, and the role of the same elements was opposite in different systems. 

Due to their excellent mechanical strength, creep resistance, and corrosion resistance, Mg-Zn-RE alloys have attracted more attention in recent years [17,18]. When studying the hot cracking behavior of Mg-Zn-RE alloys, Zhou et al. [19] investigated the solidification path and hot cracking behavior of the Mg-1Zn-*x*Y (*x* = 1, 2, 3 wt.%) alloy using a hot cracking mold. The results showed that the hot cracking volume decreased with the increasing Y element because the long-period stacking ordered (LPSO) phase is helpful to the liquid flowing and feeding for the generated cavities or micro-pores at the end of solidification. In addition to the LPSO phase, the Mg_3_Zn_3_RE_2_ phase (I-phase, quasicrystal structure) and the Mg_3_Zn_6_RE phase (W phase, cubic structure) can be obtained by adjusting the RE concentration [20]. Moreover, it has also been shown that the W phase and I-phase acting as reinforced phases were helpful in enhancing performance, especially at high temperatures [21,22]. However, the effect of the I-phase or/and W phase on the hot cracking behaviors of the casting Mg-Zn-RE alloy have been rarely reported so far.

Our group previously developed a novel Mg-Zn-Er ternary alloy possessing good creep resistance properties at high temperatures, which show potential application in aerospace industries, e.g., satellite components. In addition, the prediction of the hot cracking susceptibility of the Mg-5Zn-*x*Er alloy has been proposed [23,24]. However, the hot cracking behaviors of the alloys were unclear, which included contraction behaviors, feeding behaviors, and corresponding permeability characteristics in the solidification process. For this aim, in this work, we fabricated Mg-5Zn-*x*Er (*x* = 0.83, 1.25, 2.5, 5 wt.%) alloys with various main phase compositions (I-phase, W phase, and I-phase + W phase). The microstructure, solidification process, constrained forces, and hot cracking behaviors of the Mg-5Zn-*x*Er alloy were investigated in detail using the constrained rod casting setup. Furthermore, the relationship between microstructure and hot cracking behaviors was studied.

## 2. Experimental Method

### 2.1. Alloy Preparing

Alloys with nominal compositions of Mg-5Zn-*x*Er (*x* = 0.83, 1.25, 2.5, 5 wt.%) were prepared by melting pure Mg (99.99 wt.%), pure Zn (99.9 wt.%), and Mg-30 wt.% Er master alloy in an electric resistance furnace under the protection of a mixed gas of N_2_ and SF_6_ (volume ratio: 1000:1). The alloys were heated at 720 °C and held for 10 min in the graphite crucible. Next, the melt was stirred for 2 min to maintain the same temperature, removed the slag, and held for 15 min at a temperature of 730 °C. Then, the melt was poured into steel molds (pre-heated at 300 °C) with thin boron nitride coating for the hot cracking experiment. After the castings were completely solidified, the samples were removed to analyze the microstructures. The actual chemical compositions of the different alloys were determined three times by X-ray fluorescence spectrometry (XRF, Magix-PW2403, PANalytical, Almelo, The Netherlands), as shown in Table 1. According to our previous research [23], the main phases of the four alloys are listed in Table 1.

### 2.2. Hot Cracking Apparatus

**Figure 1:**
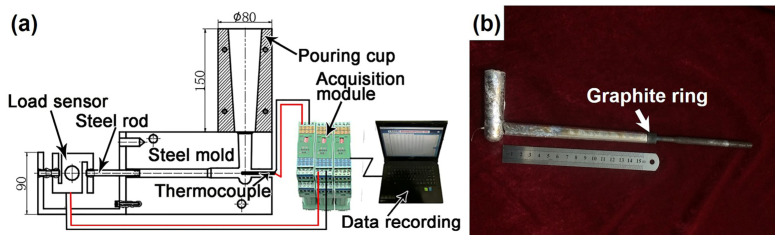
(**a**) Schematic diagram of the hot cracking mold (unit: mm); (**b**) Experimental casting.

According to the previous reports [6,15], a schematic diagram of the optimized hot cracking mold and experiment casting is shown in Figure 1. The hot cracking apparatus is composed of a constrained rod casting (CRC) steel mold, a thermocouple, a load sensor, a data acquisition system, and a pouring cup with a height of 150 mm and an outer diameter of 80 mm. When the melt was poured into the mold through a pouring cup, the melt was solidified at the end of the cavity. The load sensor was fixed with the steel screw rod (length: 120 mm and diameter: 6 mm) to maintain no movement. So the hot cracking was caused by shrinkage of the casting, and the corresponding developed force value was collected by the load sensor as a function of time. To ensure the accuracy of the experiment and eliminate the effect of friction, the graphite ring was fitted to the other end of the metallic rod in Figure 1b. Moreover, the temperature evolution during solidification was measured by the acquisition module by concatenating a thermocouple (K-type) with a diameter of 0.2 mm, and the corresponding data were recorded by the computer. The mold was pre-heated to 300 °C to ensure better filling. The chief objective of the apparatus was to acquire the evolution of the solidification process, phase reaction temperature, and contraction force to analyze the hot cracking behaviors of the alloy.

### 2.3. Microstructure Analysis

Specimens with different compositions were mechanically ground with grit SiC paper of 400#~2000#, polished using a diamond with a grain diameter of 1.5 μm and 0.5 μm, and then etched in a solution of 5 vol.% nitric acid for 10~15 s. The cross-section of the hot fracture for the alloy was carried out by Axio imager A2M optical microscope (OM, ZEISS, Jena, Germany). The hot fracture morphology of the samples in the longitudinal section was carried out by OM. A scanning electron microscope (SEM, S3400, Hitachi, Tokyo, Japan) was used to analyze the microstructure and hot cracking fracture morphology in the cross-section. The corresponding chemical composition was analyzed by an energy-dispersive spectrometer (EDS, Hitachi, Tokyo, Japan).

## 3. Results

### 3.1. Hot Cracking Samples

**Figure 2:**
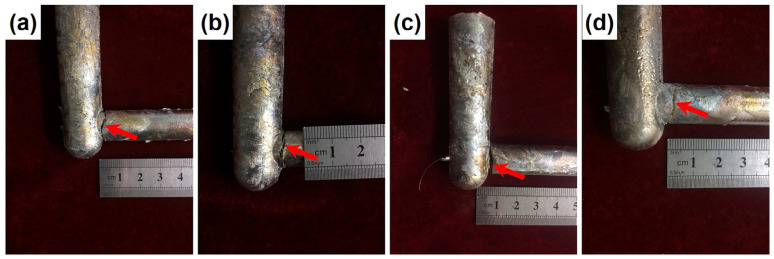
Images for hot cracking samples of Mg-5Zn-*x*Er alloys: (**a**) *x* = 0.83, (**b**) *x* = 1.25, (**c**) *x* = 2.5, and (**d**) *x* = 5.

Figure 2 shows the hot cracking samples of the Mg-5Zn-*x*Er alloy. Noticeably, the cracking for the alloy has emerged close to the sprue, which shows that the region is favorable for hot cracking due to the role of thermal contraction. There is small cracking marked with the red arrow in the Mg-5Zn-0.83Er alloy, as shown in Figure 2a. Cracking is observed in both Mg-5Zn-1.25Er and Mg-5Zn-2.5Er alloys, where the latter was more severe. Moreover, the Mg-5Zn-2.5Er alloy is completely fractured, indicating the alloy has the highest hot cracking susceptibility. On the other hand, the Mg-5Zn-5Er alloy exhibits little cracking intuitively, which indicates that the alloy shows the lowest hot cracking susceptibility among these alloys. The above results show that the hot cracking susceptibility increases as the Er content increase until 2.5 wt.% and then decreases with concentrations up to 5 wt.%.

### 3.2. Contraction Behaviors

**Figure 3:**
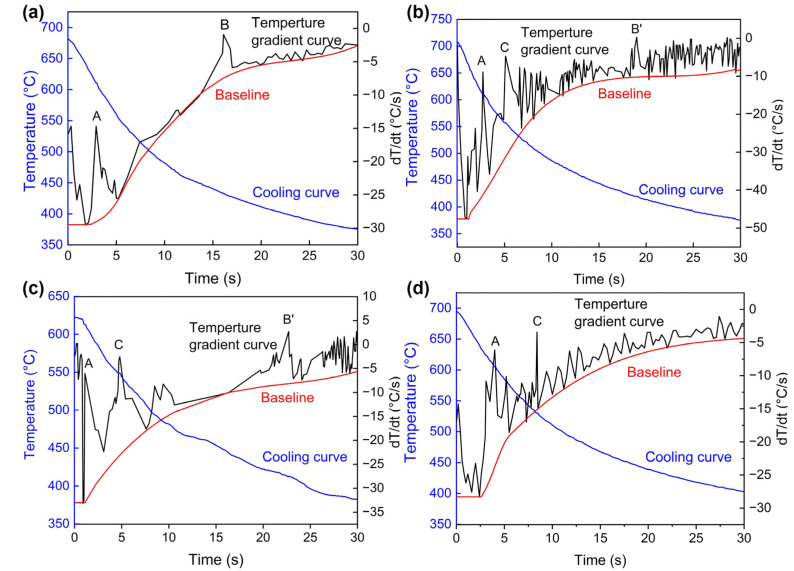
Cooling and temperature gradient curve of Mg-5Zn-*x*Er alloys in a mold temperature of 300 °C and pouring temperature of 730 °C: (**a**) *x* = 0.83, (**b**) *x* = 1.25, (**c**) *x* = 2.5, and (**d**) *x* = 5.

Hot cracking is one of the common defects in the process of casting. One of the main factors is that the cracking cannot be fed by adequate liquids in the latter stages of solidification when the solid phase fraction (*f*_s_) at this stage tends toward one [25,26]. Therefore, it is necessary to analyze the solid phase fraction for the solidification process and feeding behaviors. To obtain the *f*_s_ value, the first derivative of the cooling curve is given. According to Newton’s baseline method, *f*_s_ can be calculated by the following equation [27]:

(1) $$f_{s}=\frac{\int_{t_{L}}^{t}\left[{\left(\frac{dT}{dt}\right)}_{\mathrm{cc}}\text{−}{\left(\frac{dT}{dt}\right)}_{\mathrm{bl}}\right]dt}{\int_{t_{L}}^{t_{s}}\left[{\left(\frac{dT}{dt}\right)}_{\mathrm{cc}}\text{−}{\left(\frac{dT}{dt}\right)}_{\mathrm{bl}}\right]dt}$$

where cc is the cooling curve, bl is the baseline, *t*_L_ is the time corresponding to the initiated solidification, and *t*_s_ is the time corresponding to the solidification ending time. Figure 3 shows the cooling curves and temperature gradient curves (first derivative of the cooling curves, d*T*/d*t*) of Mg-5Zn-*x*Er alloys during solidification at a mold temperature of 300 °C and pouring temperature of 730 °C. The red curve, blue curve, and black curve are the baseline, cooling curve, and temperature gradient curve, respectively. It is assumed that it has no phase transformation in the baseline. There are obvious cooling rate changes (as indicated by points: A, B, C, and B’) in the temperature gradient curves, suggesting that different phase transformations are happening. Based on a previous study on the criteria of phase formation in the solidification process of the as-cast Mg-Zn-Er alloy [24,28,29], the distinct peak points A and B represent the nucleation of the α-Mg and non-equilibrium eutectic reaction: L → α-Mg + I-phase, which corresponds to reaction temperatures of 610.6 °C and 428.3 °C in the Mg-5Zn-0.83Er alloy, respectively. It is found that the new W phase (C point) appears in the temperature gradient curve with increasing Er content, which confirms that the reaction temperature is 557.6 °C: L → α-Mg + W phase. Similar to peak B, peak B’ also represents the formation of the I-phase, but there is a difference in the reaction process. The I-phase is formed by the peritectic reaction (L + W phase→I-phase) at the temperature of 420.2 °C (B’ point) for the Mg-5Zn-1.25Er alloy, as shown in Figure 3b. Peak A is still the formation of the α-Mg at 611.6 °C. The phase formation process of the Mg-5Zn-2.5Er alloy is the same as that of the Mg-5Zn-1.25Er alloy. The A, C, and B’ peaks appear successively in Mg-5Zn-2.5Er alloy during the cooling process. There are dividing phase transformation temperatures at 612.5 °C, 548.8 °C, and 413.0 °C, with further increases in Er content to 5 wt.%, I-phase has vanished, and the W phase is produced via the eutectic reaction at 530.8 °C: L → α-Mg + W phase. Meanwhile, the temperature with α-Mg formation is 605.0 °C, as seen in Figure 3d. 

**Figure 4:**
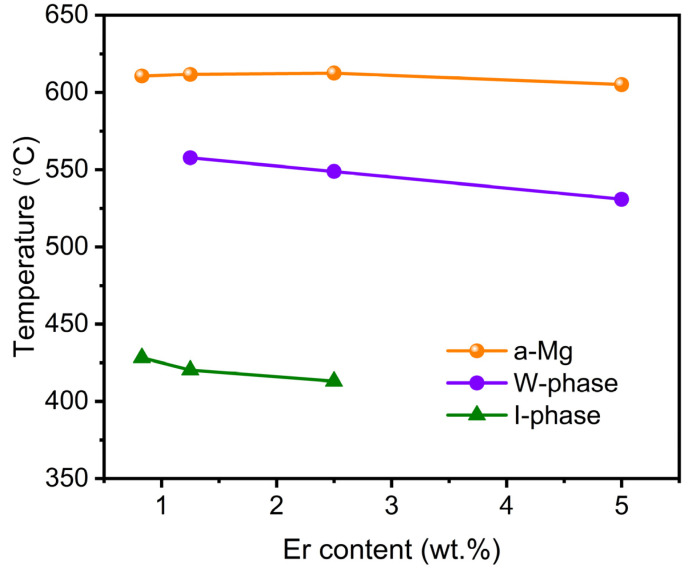
Different phase reaction temperatures of the Mg-5Zn-*x*Er alloy (*x* = 0.83, 1.25, 2.5, 5 wt.%).

The different phase reaction temperature of the Mg-5Zn-*x*Er alloy (*x* = 0.83, 1.25, 2.5, 5 wt.%) is shown in Figure 4. It indicates that the precipitation temperature of α-Mg slightly increased with the addition of Er until 2.5 wt.% and then decreased with increasing Er content to 5 wt.%. For the W phase and I-phase, the reaction temperature decreased with increasing Er concentration. In addition, the formation temperature of the I-phase (410~430 °C) is lower than that of the W phase (530~560 °C), which would affect the freezing range of the alloy.

**Figure 5:**
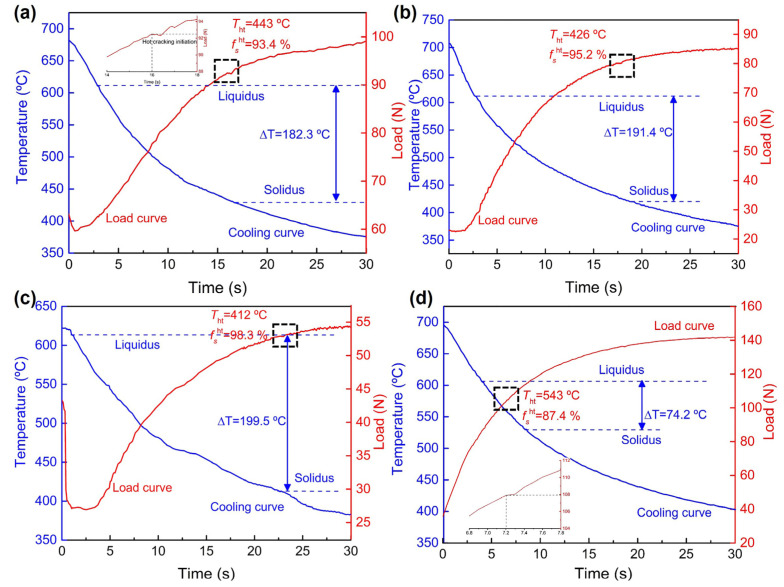
Load development as a function of time for Mg-5Zn-*x*Er alloys at a mold temperature of 300 °C and pouring temperature of 730 °C: (**a**) *x* = 0.83, (**b**) *x* = 1.25, (**c**) *x* = 2.5, and (**d**) *x* = 5.

Analyzing the hot cracking behaviors of the alloy can better understand the mechanism of hot cracking to some extent. Load development as a function of time for the Mg-5Zn-*x*Er alloys is displayed in Figure 5. For the load development curves, the contraction force abruptly drops, which is related to the stress relaxation caused by the formation of hot cracking, e.g., the dotted rectangle. Figure 5a shows the load development of the Mg-5Zn-0.83Er alloy. Since the alloy enters the mold, the force first presents a downward process due to the existing pressure on the shrinking steel rod. Afterward, the alloy begins to solidify and shrink, and then the varied value is collected by the shrinkage rod with decreasing cooling temperature. When the time reaches about 16 s, the hot cracking occurs, and the corresponding solidification temperature is 443 °C. Combined with the cooling curve and temperature gradient curve (Figure 3a), it can be estimated that the solid phase fraction at hot cracking initiation (*fs*^ht^) is 93.4%. The cooling curves have a small plateau, which is related to the released latent heat. This phenomenon suggests corresponding phase precipitation. With the moderate addition of Er (1.25 wt.%), there is W phase precipitation. As the temperature drops to 426 °C, hot cracking starts and propagates, and the corresponding time is 17.6 s. At the same time, the *f*_s_^ht^ increased to 95.4%. Figure 5c shows the curve of force development vs. time for the Mg-5Zn-2.5Er alloy. The *f_s_*^ht^ is 98.3% at hot cracking initiation, which indicates that flow liquid feeding becomes more difficult to the generated separation of dendrites in the last solidification stage because of the lack of liquid phase, and it is easy to form hot cracking. It is also observed that the load curve decreases before the initiation of solidification. When the solidification temperature is 428 °C, the corresponding hot cracking shrinkage value is 52.9 N. After hot cracking occurs, the force of shrinkage gradually tends to smooth. When the Er content reaches 5 wt.%, the freezing range of the alloy is 74.2 °C, and the corresponding time is 7.4 s. On the other hand, the load curve of the Mg-5Zn-5Er alloy has no obvious mutation, indicating that the hot cracking tendency of the alloy is the lowest. Meanwhile, the *f*_s_^ht^ is calculated to be about 87.4%, showing that there is more liquid to be fed by the eutectic reaction. 

Detailed information on the solid phase fraction and solidification temperature at hot cracking initiation are summarized in Table 2. The hot cracking temperature corresponding to the solid phase fraction is estimated by Equation (1). Among these, the force values for Mg-5Zn-1.25Er and Mg-5Zn-2.5Er alloy did not show distinct increases after hot cracking initiation, suggesting that hot cracking is more severe for this alloy than others. Similar results were also confirmed for the Mg-Ca-Zn alloy by Song et al. [16].

**Figure 6:**
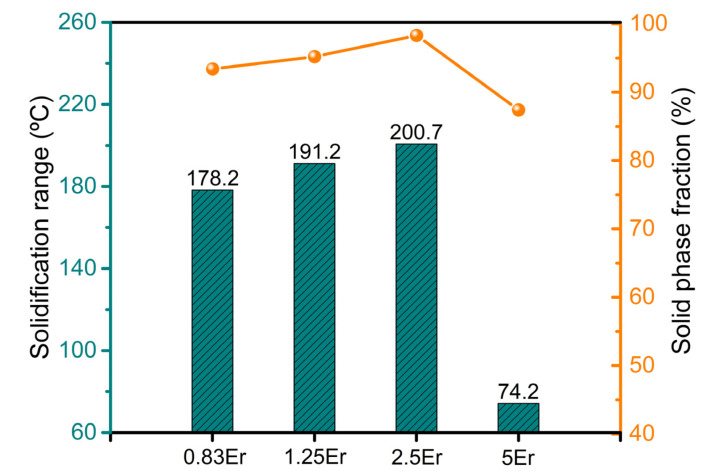
Freezing range and solid phase fraction at primary hot cracking for Mg-5Zn-*x*Er alloys (*x* = 0.83, 1.25, 2.5, 5 wt.%).

The freezing range and solid phase fraction at primary hot cracking of the Mg-5Zn-*x*Er alloy are shown in Figure 6. It can be seen that the *f_s_*^ht^ value shows some fluctuations ranging from 0.87 to 0.99, and the peak value is obtained when Er content is 2.5 wt.%. Furthermore, the freezing range and solid phase fraction at hot cracking initiation both show an “λ” curve, which shows a good correlation with the addition of Er. Therefore, the two important parameters during solidification are fine indicators of the hot cracking susceptibility of the Mg-5Zn-*x*Er alloy system.

### 3.3. Microstructures

**Figure 7:**
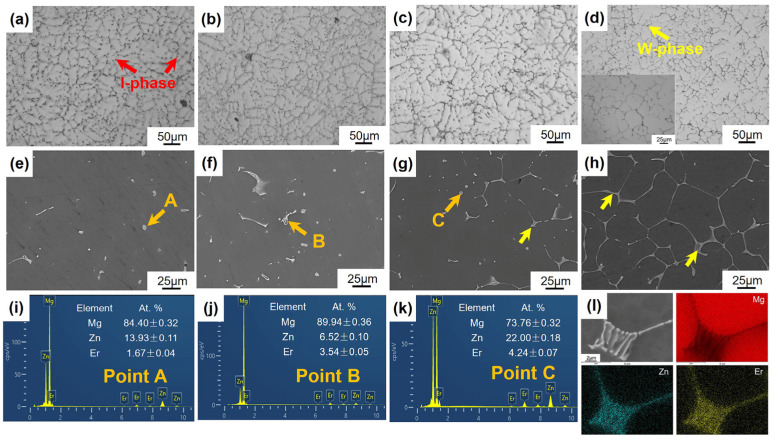
OM and SEM images of Mg-5Zn-*x*Er alloys: (**a**,**e**,**i**) *x* = 0.83, (**b**,**f**,**j**) *x* = 1.25, (**c**,**g**,**k**) *x* = 2.5, and (**d**,**h**,**l**) *x* = 5.

According to the previous research of our group, the phase composition varies with Zn/Er weight ratio in Mg-Zn-Er ternary cast magnesium alloys: (a) α-Mg + W phase (Zn/Er ≤ 0.8); (b) α-Mg + W phase + I-phase (1 ≤ Zn/Er ≤ 6); and (c) α-Mg + I-phase (6 ≤ Zn/Er ≤ 10) [24,29]. Figure 7 shows the OM and SEM images of Mg-5Zn-*x*Er alloys. Combined with Table 1, the Mg-5Zn-0.83Er alloy is composed of a gray α-Mg and black I-phase that is confirmed with the chemical composition by the EDS result (Figure 7i). The I-phase (marked by a red arrow) is mainly distributed in the grain boundary with the shape of a strip or particles. Meanwhile, a few I-phase particles irregularly appear in the grain, as shown in Figure 7a. As the Er content increased to 1.25 wt.% and 2.5 wt.%, in addition to the matrix α-Mg and quasicrystal I-phase, the W phase appeared based on the Zn/Er weight ratio [24]. The I-phase gradually decreases with increasing Er, and the morphology of the second phase at the grain boundary transforms from an irregular, discontinuous shape to a continuously distributed network microstructure, as shown in Figure 7b,c. When the Er content is 5 wt.%, the formed fishbone-like W phase (marked by a yellow arrow) is full of grain boundaries, and the interior of the grains is clean. This phenomenon indicates that the quasicrystal I-phase disappeared, as seen in Figure 7d,h. Moreover, the mapping exhibits that the W phase is mainly enriched by Er and Zn elements, as shown in Figure 7l.

**Figure 8:**
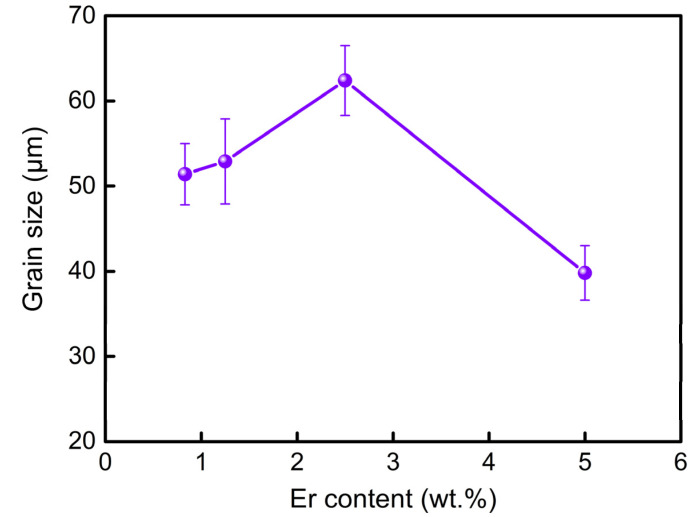
Grain size of the Mg-5Zn-*x*Er alloy (*x* = 0.83, 1.25, 2.5, 5 wt.%).

Based on ASTM standard E112-96, utilizing the line-intercept method, the grain sizes of the four alloys by measuring more than 50 grains are calculated to be 51.4 μm, 52.9 μm, 62.4 μm, and 39.8 μm, respectively, as shown in Figure 8. The grain size of the alloy increased with Er addition up to 2.5 wt.% and then decreased at 5 wt.% Er, which illustrates that grain characteristics play a crucial role in the hot cracking susceptibility of the Mg-5Zn-*x*Er alloy. Detailed analysis will be investigated in the discussion.

### 3.4. Hot cracking Fracture

**Figure 9:**
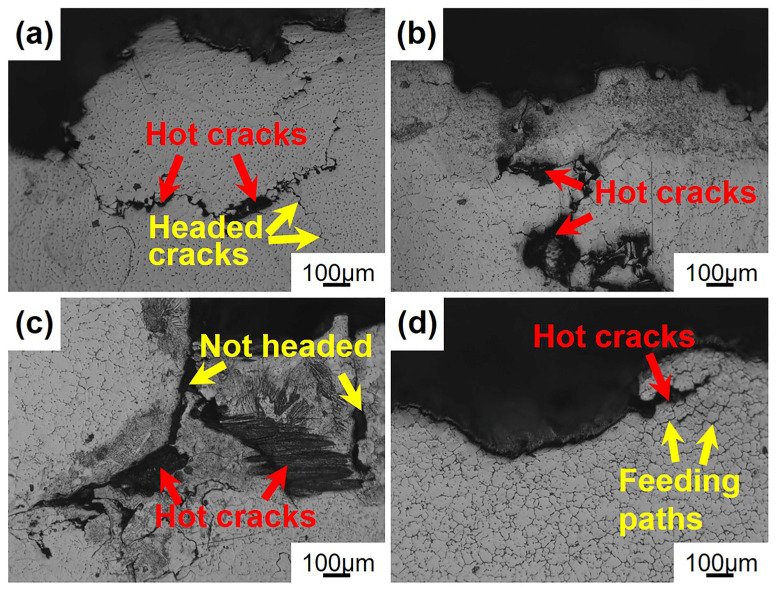
Hot cracking fracture morphology of the Mg-5Zn-*x*Er alloys in the longitudinal section: (**a**) *x* = 0.83, (**b**) *x* = 1.25, (**c**) *x* = 2.5, (**d**) *x* = 5.

Figure 9 shows the hot crack fracture morphology of the Mg-5Zn-*x*Er alloy in the longitudinal section. It can be seen from Figure 9a that there are traces of healed hot cracks (yellow arrows) around the hot fracture in the Mg-5Zn-0.83Er alloy. However, a portion of the hot crack did not effectively heal, contributing to hot cracks expanding and eventually generating larger hot cracks. With increasing Er content (1.25 wt.%), the hot cracking fracture area increases, indicating that the hot cracking susceptibility of the alloy increases in Figure 9b. The fracture area of the Mg-5Zn-2.5Er alloy is the largest, as shown in Figure 9c. The feeding is less (*f_s_*^ht^ = 98.3%), which cannot be compensated for by the cracks generated, which leads to the larger-area hot cracks. When the Er content increases to 5 wt.%, the existing eutectic liquid can effectively feed the cracks by eutectic reaction, which inhibits the initiation and propagation of hot cracking, greatly reducing the hot cracking susceptibility in the Mg-5Zn-5Er alloy, as shown in Figure 9d.

**Figure 10:**
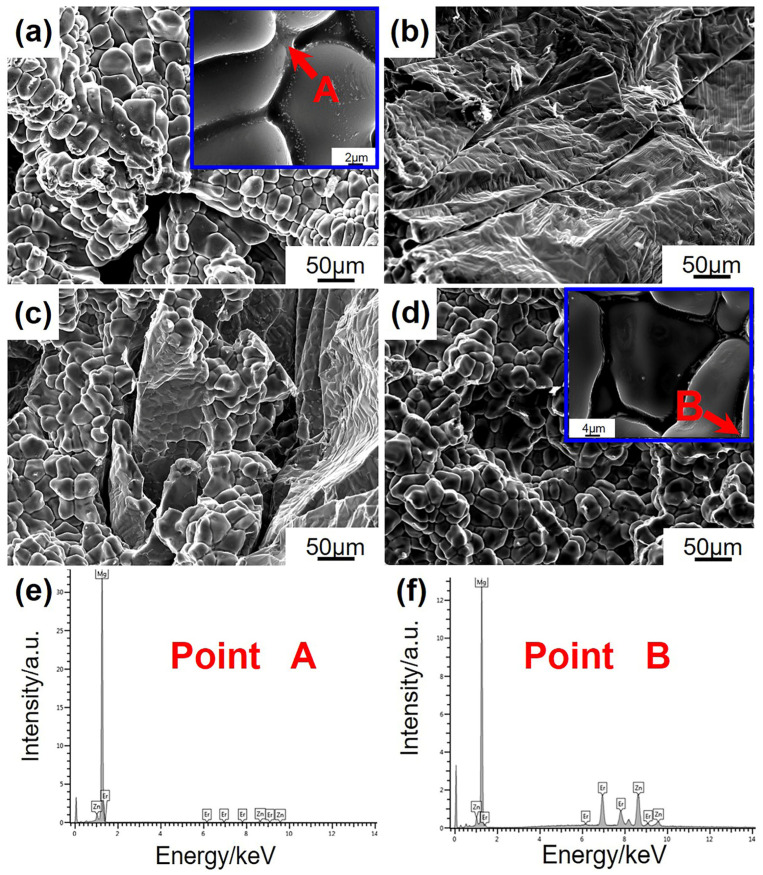
Hot cracking fracture morphology of Mg-5Zn-*x*Er alloys in the cross-section: (**a**,**e**) *x* = 0.83, (**b**) *x* = 1.25, (**c**) *x* = 2.5, and (**d**,**f**) *x* = 5.

SEM observations were analyzed on the hot cracking fracture morphology of the Mg-5Zn-*x*Er alloys in the cross-section, as shown in Figure 10. There is a liquid film on the surface, which is the remaining liquid phase near the solidus. The fracture dendrites are smooth and large for the Mg-5Zn-0.83Er alloy seen in Figure 10a. During the cooling process of the sample, the liquid phase fraction decreases, which leads to the liquid film gradually decreasing and ultimately disappearing. Combined with Table 2, the solid phase fraction at hot cracking initiation is relatively low, so there are more feeding liquids. Consequently, the alloy has good hot cracking resistance. With the addition of 1.25 wt.% Er, the liquid film of the alloy is thin and discontinuous, which cannot withstand shrinkage forces (Table 2). Therefore, it is unfavorable for the hot cracking susceptibility of the Mg-5Zn-1.25Er alloy seen in Figure 10b. Figure 10c displays the hot cracking fracture of the Mg-5Zn-2.5Er alloy. The liquid film is in an intermittent morphology, meaning that the liquid phase around the grains is less in the solidification process. Accordingly, the intergranular strength is limited, which fails to resist the applied shrinkage force, and the Mg-5Zn-2.5Er alloy has the highest hot cracking susceptibility. When the Er content is 5 wt.%, the number of eutectic liquids in the fracture of Mg-5Zn-5Er alloy increases significantly, which allows a large amount of liquid phase to feed the formed cracks. Moreover, remarkable fractured bridges that connected the two grains can be observed in Figure 10d. With the same number of flow feeding channels, at a lower solid phase fraction and higher remaining liquid phase, the feeding effect is improved, and hot cracking susceptibility of the Mg-5Zn-5Er alloy is reduced. The corresponding solid phase fraction of the Mg-5Zn-2.5Er alloy is the lowest, which means that the alloy has the highest hot cracking susceptibility in Table 2. The EDS results of points A and B are summarized in Table 3. The Zn/Er ratio value is ~6 with the composition analysis of the fracture dendrite in the Mg-5Zn-0.83Er alloy, marked with a red arrow in Figure 10a, which indicates that the liquid phase is the feeding I-phase. Combined with the fishbone-like morphology in Figure 7d, it can be deduced that the feeding liquids of the Mg-5Zn-5Er alloy are W phase, as confirmed with the Zn/Er ratio of 2.1.

## 4. Discussion

### 4.1. Freezing Range and Feeding Behaviors

As shown in Table 2, due to the different temperatures corresponding to the different stages of hot cracking initiation, it can be deduced that the hot cracking behaviors of the alloy are distinguishable, which is mainly related to the addition of Er. Admittedly, the solidification range of the alloy has significant importance on the hot cracking behaviors. It can be seen that the solidification temperature range presents an “λ” type, which affects the hot cracking susceptibility of the alloy in Figure 6. If one assumes that the solidus and liquidus of the alloy are *T*_s_ and *T*_L_, respectively (both are straight lines, equilibrium solidification) and the composition of the alloy is *C*_0_, the solidification range can be expressed by the following Equation (2) [30]:

(2) $$\Delta T_{f}{=mC}_{0}\frac{{1-k}_{0}}{k_{0}}$$

where Δ*T_f_* is the freezing range of the alloy when the composition of the alloy is *C*_0_, *m* is the slope of the liquids, and *k*_0_ is the equilibrium partition coefficient. Assume the following Equation (3):

(3) $$m\frac{{1-k}_{0}}{k_{0}}{=r}_{f}$$

Therefore, Equation (2) becomes Equation (4):

(4) $$\Delta T_{f}{=C}_{0}r_{f}$$

Equation (4) illustrates that the Δ*T_f_* value is affected by the composition *C*_0_. During the actual solidification of the alloy, the atomic diffusion process and instability of the solid–liquid interface cause non-equilibrium solidification of the alloy, leading to changes in the *m* value. According to the Scheil non-equilibrium model, it is known that the value of *k*_0_ will change with changes in Er element content, which impacts Δ*T_f_* [31]. In general, the hot cracking zone of the alloy is proportional to the freezing range. The solidification temperature range of the Mg-5Zn-*x*Er alloy shows a trend of first increasing up to 2.5 wt.% Er and then decreasing in 5 wt.%, as shown in Figure 6 and Table 2. Therefore, the corresponding hot cracking susceptibility of the alloy first increased up to 2.5 wt.% and then decreased. This means that the hot cracking susceptibility of the Mg-5Zn-2.5Er alloy is highest, and the Mg-5Zn-5Er alloy is lowest. The above results match well with the macroscopic samples of the hot cracking in Figure 2.

### 4.2. Permeability of the Mushy Zone

**Figure 11:**
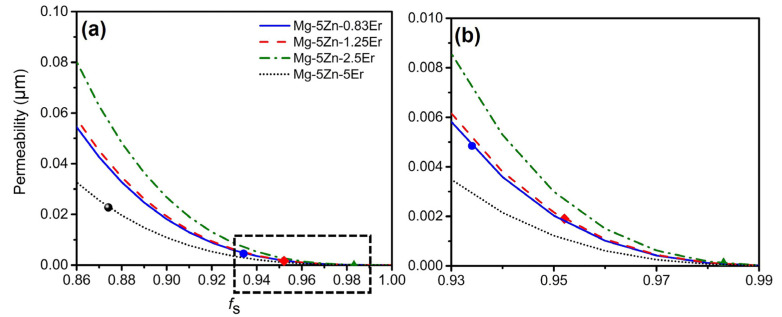
(**a**) Permeability of the mushy zone for the Mg-5Zn-*x*Er alloy (*x* = 0.83, 1.25, 2.5, 5 wt.%) at hot cracking initiation; (**b**) The magnified image in the selected rectangle in (**a**).

Song et al. [16] pointed out that the grain size of the Mg-0.5Ca-4Zn-Zr (wt.%) alloy is lower than that of the Zr-free alloy at the same mold temperature, which increased the hot cracking resistance of the alloy. For the Mg-5Zn-*x*Er alloy, the effect of grain refinement (Figure 7 and Figure 8) on the hot cracking susceptibility can be explained by the permeability. The permeability is determined by the following Kozeny–Carman in Equation (5) [32]:

(5) $$K=\frac{\rho^{2}}{180}\frac{{\left({1-f}_{s}\right)}^{3}}{{\;f}_{s}{}^{2}}$$

where *ρ* is grain size, *f_s_* is solid phase fraction, and *K* is the permeability of the mushy zone. Substituting the corresponding parameters into Equation (5), the relationship between the solid phase fraction at hot cracking initiation and the permeability of the alloy can be obtained, as shown in Figure 11. The permeability of the Mg-5Zn-2.5Er alloy is the lowest, and the Mg-5Zn-5Er alloy is the highest. The corresponding results suggest that liquid phase feeding of the Mg-5Zn-5Er alloy in the mushy zone is greatest, which decreases the hot cracking susceptibility. In the solidification stage, the solidification shrinkage and thermal contraction can be properly compensated by the flow liquid phase. Then, in the case of the restricted liquid or limited feeding ability during solidification, the cavities are formed, which is directly connected with the feeding rate (*f_e_*). Furthermore, the feeding rate is affected by *K* and has a proportional relationship with *K*: *f_e_* ∝ *K* [33,34]. A faster feeding rate and a larger amount of the liquid phase contribute to the effective feeding to the voids and form micro-pores. The improved feeding ability was further verified by the hot cracking fracture (Figure 9 and Figure 10).

Grain refinement can delay the feeding of the hot cracking in the mushy zone and increase the feeding time, which effectively suppresses the propagation of the hot cracking and reduces the hot cracking susceptibility of the alloys [6]. Meanwhile, it is considered that the columnar grains are unfavorable, which will promote the initiation and propagation of hot cracking [35]. Considering the relationship between hot cracking and solidification shrinkage based on the stress-based criteria, the strength generated by the grains cannot resist the shrinkage force in the semi-solid alloy at the end of solidification, bringing about the hot cracking of the alloy [5]. The deformation of the semi-solid alloy caused by the solidification shrinkage will be better coordinated in the finer grains. The local force will be lower, which is beneficial to resisting the occurrence of hot cracking and reducing the hot cracking susceptibility of the alloy [36,37]. In this work, the Mg-5Zn-5Er alloy has the lowest hot cracking susceptibility due to its finest grain size of 39.8 μm.

Moreover, the Mg-5Zn-*x*Er alloy with different Er contents forms W phases and/or I-phases during solidification. Combined with different phase reaction temperatures in Figure 4, the I-phase mainly precipitated at 410–430 °C via the eutectic reaction (L→α-Mg + I-phase) or the peritectic reaction (L + W phase→I-phase). The phase transformation temperature of the W phase (530–560 °C) is higher than that of the I-phase. When Er content is 1.25 wt.% or 2.5 wt.%, the W phase begins to precipitate by the eutectic reaction (L→α-Mg + W phase). In addition, the remaining liquids in the alloy still take in the subsequent peritectic reaction to generate the I-phase (L + W phase→I-phase), which causes the lower liquids in the peritectic reaction process. Hence, the lack of surplus liquids and the wide freezing range can lead to a higher tendency for hot cracking, which was demonstrated by the solid phase fraction at hot cracking initiation (Table 2). On the other hand, when the Er element increases to 5 wt.%, the freezing range of the Mg-5Zn-5Er alloy is reduced (74.2 °C). On the other hand, the W phase generated via the eutectic reaction (L→α-Mg + W phase) acts as an effective liquid (*f_s_*^ht^ = 87.4%) to feed the formed cavities or micro-pores, which decreases the hot cracking susceptibility of the alloy. Based on this information, it can be confirmed that the Mg-5Zn-2.5Er alloy has maximal hot cracking susceptibility, and the Mg-5Zn-5Er alloy exhibited minimal hot cracking susceptibility. This is consistent with previous results generated by the optimizing Rappaz–Drezet–Gremaud model [23].

In this study, the hot cracking behaviors of Mg-Zn-Er alloys with different Er contents were studied. Nevertheless, this is a qualitative estimation of the hot cracking process; for example, the relationship between temperature, solid phase fraction, and force relationship at hot cracking initiation. Therefore, quantitative estimation should be performed to predict and evaluate the hot cracking process. Moreover, cast fluidity is also an important performance for the application of the as-cast Mg-Zn-Er alloy, and the corresponding results will be reported separately. This work provides available guidance for the design of hot cracking susceptibility in the Mg-Zn-RE alloy system.

## 5. Conclusions

To understand the hot cracking behaviors of Mg-Zn-Er alloys with different Er contents, the contraction behaviors, feeding behaviors, and corresponding permeability characteristics were investigated. The following conclusions can be drawn:

(1) The freezing range and solid phase fraction at hot cracking initiation increase with increasing Er content up to 2.5 wt.% and then decrease with concentrations up to 5 wt.%. The Mg-5Zn-5Er alloy exhibits the highest liquid phase fraction and reduced freezing range, contributing to the decreased hot cracking tendency, which shows minimal hot cracking susceptibility. Conversely, the Mg-5Zn-2.5Er alloy exhibits the maximal hot cracking susceptibility;
(2) The Mg-5Zn-*x*Er alloys with different Er contents form the W phase and/or I-phase during solidification. The I-phase of the Mg-5Zn-0.83Er alloy is formed by the eutectic reaction. When the Er content is 1.25 wt.% or 2.5 wt.%, the W phase precipitates first, and the remaining liquids still contribute to the subsequent peritectic reaction to generate the I-phase. The lack of surplus liquids leads to a higher hot cracking tendency. For the Mg-5Zn-5Er alloy, the more effective liquids by the eutectic reaction (L → α-Mg + W phase) and high phase precipitation temperature lead to the lowest freezing range;
(3) The Mg-5Zn-5Er alloy exhibits the best permeability of the mushy zone due to the refined grain size, which is beneficial to feed the emerging cavities and micro-pores. Meanwhile, a large number of eutectic phases at the fracture would heal the cracking, which increases the hot cracking resistance of the alloy.

## Figures and Tables

**Table 1:** Actual composition and main phases of the Mg-5Zn-*x*Er alloys (*x* = 0.83, 1.25, 2.5, 5 wt.%).

Alloy	Zn (wt.%)	Er (wt.%)	Mg (wt.%)	Main Phases
Mg-5Zn-0.83Er	5.3 ± 0.3	0.8 ± 0.1	Bal.	α-Mg + I-phase
Mg-5Zn-1.25Er	5.0 ± 0.1	1.1 ± 0.3	Bal.	α-Mg + I-phase + W phase
Mg-5Zn-2.5Er	5.1 ± 0.3	2.4 ± 0.1	Bal.	α-Mg + I-phase + W phase
Mg-5Zn-5Er	5.1 ± 0.2	5.3 ± 0.2	Bal.	α-Mg + W phase

**Table 2:** Measurement data of the Mg-5Zn-*x*Er alloys (*x* = 0.83, 1.25, 2.5, 5 wt.%) from Figure 3 and Figure 5. *f_s_*^ht^: solid phase fraction at hot cracking initiation, *T*_L_: liquidus temperature, *T*_s_: solidus temperature, ΔT: freezing range, *T*_ht_: solidification temperature at hot cracking initiation.

Alloy	*f_s_*^ht^ (%)	*T*_L_ (°C)	*T*_s_ (°C)	ΔT (°C)	*T*_ht_ (°C)
Mg-5Zn-0.83Er	93.4	610.6	428.3	182.3	443
Mg-5Zn-1.25Er	95.2	611.6	420.2	191.4	426
Mg-5Zn-2.5Er	98.3	612.5	413.0	199.5	412
Mg-5Zn-5Er	87.4	605.0	530.8	74.2	543

**Table 3:** EDS results of Points A and B in Figure 10.

Point	At. %			Zn/Er Ratio	Phase
	Mg	Zn	Er		
A	97.72	1.96	0.32	6.1	I-phase
B	64.81	23.78	11.41	2.1	W phase

## Data Availability

Data are contained within the article and can be requested from the corresponding author.
